# Culicidae Community Composition and Temporal Dynamics in Guapiaçu Ecological Reserve, Cachoeiras de Macacu, Rio de Janeiro, Brazil

**DOI:** 10.1371/journal.pone.0122268

**Published:** 2015-03-27

**Authors:** Jeronimo Alencar, Cecilia Ferreira de Mello, Anthony Érico Guimarães, Hélcio R. Gil-Santana, Júlia dos Santos Silva, Jacenir R. Santos- Mallet, Raquel M. Gleiser

**Affiliations:** 1 Instituto Oswaldo Cruz (Fiocruz), Laboratório de Diptera, Manguinhos, Rio de Janeiro, Brazil; 2 Instituto Oswaldo Cruz (Fiocruz), Laboratório de Transmissores de Leishmanioses, Manguinhos, Rio de Janeiro, Brazil; 3 Centro de Relevamiento y Evaluación de Recursos Agrícolas y Naturales-Instituto Multidisciplinario de Biología Vegetal (Consejo Nacional de Investigaciones Científicas y Técnicas—Universidad Nacinal de Córdoba, CONICET-UNC), Facultad de Ciencias Agropecuarias, and Cátedra de Ecología, Facultad de Ciencias Exactas, Físicas y Naturales, Universidad Nacional de Córdoba, Córdoba, Argentina; Universidad Nacional Autonoma de Mexico, MEXICO

## Abstract

A temporal observational study was conducted of the Culicidae fauna in a remnant area of Atlantic Forest within a private reserve (Guapiaçu Ecological Reserve-REGUA) presenting typical vegetation cover of dense rain forest, with some patches recovering a floristic composition similar to that of the original community. Research was carried out to analyze the influence of climatic factors (mean monthly temperature, rainfall, and air relative humidity) on the temporal dynamics of the mosquito communities that occur in the reserve. The completeness of the mosquito inventory was assessed with individual-based rarefaction-extrapolation curves. Differences in species composition between sites and months were tested with PERMANOVA. True diversities of orders 0, 1, and 2 (effective numbers) were estimated and compared between sites, months, and years. Multiple stepwise regressions were used to assess relationships between climatic variables, measures of diversity, and abundances of the most common species. There were significant interactive effects between year and site on measures of diversity. However, diversity estimates showed little variation among months, and these were weakly correlated with climatic variables. Abundances of the most common species were significantly related to temperature or relative humidity, but not rainfall. The presence of mosquito species known to be vectors of human diseases combined with an intermittent flow of visitors to the study area suggests there is a risk of disease transmission that warrants further monitoring.

## Introduction

The Atlantic Forest of South America is ranked among the world’s top biodiversity hotspots. Stretching along the Atlantic coastline from northeast Brazil to Uruguay, only 7% of the original forest area still remains. It has been extensively modified, and within Brazil, reduced to 7.8% of its original cover. This ecosystem is one of the most endangered in the world, second only to the severely threatened forests of Madagascar, off the east coast of the African continent [1]. About 5–12% of the remaining area of the Atlantic Forest, including legally protected areas, is composed of relatively small forest fragments [2].

A rich diversity of species from the Culicidae family (Order: Diptera) with considerable spatial variability in composition has been recorded in the Atlantic Forest. For example, 22 species were collected from bromeliads belonging to the genera *Nidularium* and *Vrisea* in Serra do Mar [3]; 91 taxa were recorded in degraded and remnant forests in the Municipality of São Paulo, Brazil [4], and 31 mosquito species belonging to 12 genera were collected in Nova Iguaçu Natural Park, Rio de Janeiro [5]. The Atlantic forest is topographically complex, which creates a diverse array of microclimates and environmental conditions that may affect the availability and suitability of mosquito habitats, resulting in substantial spatial variation in Culicidae assemblages within this ecoregion [6], [3], [5].

The study of mosquitoes in natural areas is of considerable importance due to their role in pathogen transmission to humans and other vertebrates [7] and the potential to identify as yet unknown habitats of these disease vectors [8]. Knowledge of mosquito community composition is of fundamental importance in areas where the environment has suffered or is suffering anthropic disturbances. The composition and diversity of mosquito communities may influence disease transmission, either decreasing disease risk through mechanisms such as competition for hosts among vector and non-vector species [9], or facilitating the spread of disease due to factors such as nested ectoparasite-vector host networks [10]. Improved knowledge of culicid populations can only be achieved if the systematics and ecology of the group are both studied. The aim of this study was to document temporal changes in the distribution of mosquito species from the Guapiaçu Ecological Reserve, Cachoeiras de Macacu, Rio de Janeiro, Brazil, and investigate the influence of seasonally variable climatic factors (temperature, relative humidity and rainfall) on species abundances and diversity.

## Materials and Methods

### Ethics statement

All research was performed in accordance with scientific license number 34911 provided by SISBIO/IBAMA for the capture of culicids throughout the Brazilian national territory.

### Study area

Mosquito collections were made in the Guapiaçu Ecological Reserve (REGUA), a Private Natural Heritage Reserve that was created in 1996 and contains about 7,385 ha of dense rain forest. The reserve has a great wealth of pioneer species in its lower and peripheral areas, culminating in climax communities in its highest parts. Altitude ranges from 30 to 2000 m above sea level, and part of the reserve area, particularly that above 700 m, is covered by largely unmodified forests hosting a large diversity of plants and animals. Over 80% of the reserve is above 400 m and is part of the Three Peaks State Park, located in the central corridor of the Serra do Mar, Atlantic Forest biome.

Vegetation in REGUA is characterized as dense rain forest, consisting of three vegetation types: the lower part consists of dense alluvial rain forest and pasture that has been reverting to forest for the past seven years; coastal plains, lowlands, and lower mountain slopes with dense rain forest and areas that have been reforested for 3–5 years; and dense submontane and montane rain forest covering the rugged mountainous areas [11]. The latter holds mature forest that was used as a reference in reforestation programs. The soils of the study area are classified as Fluvisol, Oxisol, and Cambisol on the plains, gently undulating slopes, and hills, respectively. In the large Guapi-Macacu watershed (Guapiaçu and Macacu rivers), Pinheiro [12] suggested the occurrence of other soil classes. In hilly areas there is a predominance of red-Oxisols; areas with rugged, rocky outcrops have Haplic Cambisols and Entisols; yellow and red-yellow Argisols and Haplic Planosols can be found in alkaline massifs; and Fluvic Neossolos and Gleysols predominate in hydromorphic areas.

**Fig 1:**
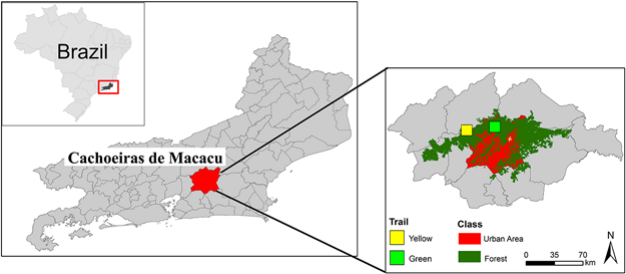
a: Location in Brazil; b: State of Rio de Janeiro; c: Guapiaçu Ecological Reserve (REGUA), with sampling sites A and B marked.

Two sampling sites were established: Site A was located in the lower parts of the study area in flat terrain presently covered by pasture and 7-year-old regenerated forest. It is adjacent to the reserve administration, in an area featuring wetlands that were revegetated in 2005. The seedlings used for revegetation were mostly produced with genetic material sourced from forest remnants within the reserve itself, supplemented with seedlings from nurseries in the area. Native species were planted with random distributions. Random mixtures of pioneer, early, and late secondary, and climax species were used, with pioneers making up the largest proportion. This was done to avoid spatial homogeneity in community composition. Exotic fruiting species were also planted in order to attract frugivorous birds. Site B was located in the submontane zone, in the highest part of the study area, overlooking hilly and rugged terrain. The forest is dense and highly diverse submontane and montane rain forest, comprising three strata: emergent trees (reaching to about 45 m high), the main canopy (5–10 m in height), and understory vegetation [13]. Geographical coordinates of the sampling sites were obtained using a Garmin GPSmap 60CS GPS. Maps were prepared in Arcview10 and edited in Adobe Photoshop CS5 and CorelDraw X5. The sampling locations are shown in Fig. 1.

Culicid sampling took place once every two months (at approximately the same day of the month) for two years, from February 2012 to January 2014, for three consecutive nights, with each sampling running from 6:00pm to 10:00am. Mosquitoes were collected using two types of light trap: automatic CDC (Center for Disease Control) and Shannon [14], with the aid of an oral suction tube. Light traps are a standard method for sampling mostly crepuscular and nocturnal mosquitoes. Although there is variation in the degree to which different mosquito species (or populations of a species from different geographic regions) are attracted to light traps, this method is considered to provide the least biased samples of aerial mosquito populations [15]. For data analysis, captures from both trap types were pooled.

Species determinations were performed by direct observation of morphological characters using a stereomicroscope (ZEISS Stemi SV6) and, when necessary, optic microscope examination of key morphological characteristics of individuals mounted on microscope slides (e.g., male genitalia). Species identifications were based on dichotomous keys by Lane [16], Faran and Linthicum [17], Consoli and Lourenço-de-Oliveira [18], and Forattini [19]. For species of the Aedini tribe, we followed the policy of the Journal of Medical Entomology and considered *Ochlerotatus* to be a subgenus of *Aedes* [20], as opposed to the taxonomy proposed by Reinert [21], which endows *Ochlerotatus* with genus status. All mosquito samples were then deposited in the Entomological Collection of the Oswaldo Cruz Institute, under the title “Atlantic Forest Collection”.

Monthly mean measurements of air relative humidity, temperature (maximum, minimum, and average offset) and monthly rainfall were obtained from the National Meteorology Institute of Brazil (INMET). For each site and sampling date, variations in temperature and relative humidity were measured every hour using a thermo-hygrometer (Oregon Scientific, RMR132HG), fixed 1 m above ground level. Comparisons between field collected data and data provided by INMET did not show significant differences, and thus INMET data were used in the analysis.

### Data analyses

To verify the completeness of the mosquito inventory, a species accumulation curve was generated for each site, using individual-based interpolation (rarefaction) and extrapolation (up to double the lowest number of individuals recorded at either site) from reference samples (total number of individuals collected at each site) using the multinomial model (S(est)) [22] in EstimateS software [23] (i.e., the expected number of species represented among m individuals, given the reference sample). Richness estimates, standard errors, and 95% confidence intervals were calculated. Further, considering that the performance of richness estimators varies among data and cases [24], [25], the following well-known species richness estimators (diversity of order 0) were assessed using SPADE software [26]: Chao1-bc (a bias-corrected form of Chao1, [27]) and ACE-1 (a modified non-parametric abundance-based coverage estimator for highly heterogeneous communities [28]). Rare species were defined as those for which fewer than 10 individuals were collected. The number of species common to both sites and the number of species expected if sample size was increased were estimated using the concept of sample coverage [29] and SPADE software (200 bootstrap replications used to obtain the standard error estimate). The squared coefficient of variation of species abundance (CV) was estimated to characterize the degree of heterogeneity among species abundances; when all species have equal abundances, CV is zero, and is positively correlated with the degree of heterogeneity [30].

To test for differences in species composition between sampling sites and among months, a non-parametric multivariate analysis of variance (PERMANOVA) with 10,000 permutations based on Bray-Curtis distances was used. To visualize differences in multivariate patterns among observations, non-metric multidimensional scaling (nMDS) was performed on the Bray-Curtis distances (Past software, [31]).

In addition to species richness (diversity of order 0), other measures of species diversity were estimated: Shannon’s index and associated effective number of species (diversity of order 1, or Shannon diversity, based on the Chao & Shen estimator [30]), and Simpson’s index and associated effective number of species (diversity of order 2, or Simpson diversity, based on a minimum variance unbiased estimator—MVUE). Differences between sites were assessed with t-tests.

Generalized linear models (GLMs) were used to assess differences in abundance and diversity (for each order of diversity: 0, 1, or 2) between sites and years (2012 and 2013), and among months (February, April, June, August, October, and December) (Infostat software [32]). Mosquito abundance, Shannon Diversity (diversity of order 1), and Simpson Diversity (diversity of order 2) were assumed to be Poisson log distributed, while species richness (diversity of order 0) was assumed to be Gaussian distributed. The threshold for assessing significant differences was set at p < 0.05.

Relationships between average monthly climatic variables from the same or previous month as mosquito collection (rainfall, maximum, and minimum temperature, and relative humidity) and abundances and measures of diversity of the eight most abundant species (*Aedes (Ochlerotatus) scapularis* (Rondani, 1948), *Anopheles (Nyssorhynchus) albitarsis* Lynch Arribálzaga, 1878, *Culex* (*Melanoconion*) *bastagarius* Dyar and Knab, 1906, *Cx*. (*Culex*) *declarator* Dyar and Knab, 1906, *Cx. (Cux.) usquatus* Dyar, 1918, *Coquillettidia venezuelensis* (Theobald, 1912), *Mansonia (Mansonia) titillans* (Walker, 1848), and *Ma. (Man.) wilsoni* (Barreto and Coutinho, 1944)) were assessed with multiple stepwise regression (Stepwise in InfoStat software), with p ≤ 0.15 as the criterion for retaining variables.

## Results

A total of 3,289 individual mosquitoes were collected during the two year sampling period, of which 3,170 (96.4%) were identified to the species level (Table 1). The identities of the remaining 3.6% could only be determined to the genus level because of damage to the relevant morphological characteristics. Most specimens were collected using CDC light traps (3,092); only 197 individuals were collected with Shannon traps, and none of these at site B. Table 2 summarizes the species collected by site and trap type. The mosquitoes captured belonged to 48 species from 14 genera: *Aedeomyia* (0.4%), *Aedes* (5.22%), *Anopheles* (5.7%), *Coquillettidia* (10.9%), *Culex* (58.9%), *Haemagogus* (0.1%), *Limatus* (1%), *Mansonia* (11.3%), *Psorophora* (0.3%), *Rhunchomyia* (1%), *Sabethes* (0.2%), *Trichoprosopon* (0.2%), *Uranotaenia* (3.4%), and *Wyeomyia* (0.8%). The most frequently captured species was *Cx. bastagarius* (21.7%), followed by *Cx. usquatus* (17.4%), *Cx. declarator* (15.6%), and *Ma. titillans* (5.9%).

**Table 1:** Absolute values (N) of mosquito species collected in the Guapiaçu Ecological Reserve, Cachoeiras de Macacu, Rio de Janeiro, Brazil, in the period from February 2012 to January 2014.

Year	2012							2013							2014	
Month	Feb	Apr	Jun	Aug	Oct	Nov	Dec	Feb	Apr	May	Jun	Aug	Oct	Dec	Jan	Total
*Ad. (Ady.) squamipennis* (Lynch Arribalzaga), 1878	1						10			1			1	1	1	15
*Ae. (Och.) fluviatilis* (Lutz), 1904	1												1	1		3
*Ae. (Och.) rhyacophilus* Costa Lima, 1933			1			1			17							19
*Ae. (Och.) scapularis* (Rondani),1948	11				4	7		23	38	6	4	2			9	104
*Ae. (Och.) serratus* (Theobald), 1901	22					1		1	27	1						52
*Ae. (Pro.) terrens* (Walker),1856			14							1		1				16
*Ae. (Stg.) albopictus* Skuse, 1984							1		1		1					3
*An. (Nys.) albitarsis* Lynch-Arribalzaga, 1878						7	1	1	63		13	23	7	2	6	123
*An. (Nys.) evansae* (Brethes), 1926					1				37	1		6	3			48
*An. (Nys.) minor* Da Costa Lima, 1929						2										2
*An. (Nys.) triannulatus* (Neiva and Pinto), 1922					1			1	12			9				23
*Cq. (Rhy.) albicosta* (Peryassu), 1908	2					2			36	2						42
*Cq. (Rhy.) chrysonotum* (Peryassu), 1922										1						1
*Cq. (Rhy.) juxtamansonia* (Chagas), 1907	16				1	4	1		41	10		19	2			94
*Cq. (Rhy.) fasciolata* (Lynch Arribalzaga), 1891	4		1		1	9			56	10		19				100
*Cq. (Rhy.) venezuelensis* (Theobald, 1912)					2		1		36	20		38	25	17	1	140
*Cx. (Cux.) bidens* Dyar, 1922								4		6						10
*Cx. (Cux.) declarator* Dyar and Knab, 1906	29	7	8	4	6	74	59	13	218	30	8	36	19			511
*Cx. (Cux.) quinquefasciatus* Say, 1823							2	1					2			5
*Cx. (Cux.) usquatus* Dyar, 1918	38	14	6	4	5	87	40	8	256	30	17	53	15			573
*Cx. (Mcx.) imitator* Theobald, 1903			2	1												3
*Cx. (Mel.) bastagarius* Dyar and Knab, 1906					2		88		206	5	60	243	110	313	76	1103
*Cx. (Cux.)* sp1				6			1	43	4				37	20	2	113
*Cx. (Cux.)* sp2							4	21					3	20	2	50
*Hg. (Hag.) capricornii* Lutz, 1904			1													1
*Hg. (Hag.) leucocelaenus* (Dyar and Shannon),1924		1														1
*Li. durhamii* Theobald, 1901						7			4			6				17
*Li. flavisetosus* Oliveira Castro, 1935							8						7			15
*Ma. (Man.) indubitans* Dyar and Shannon, 1925														3		3
*Ma. (Man.) titillans* (Walker), 1848	12					10			165				6		3	196
*Ma. (Man.) wilsoni* (Barreto and Coutinho), 1944					1		10	10	127	2	7	18	4			179
*Ps. (Jan.) ferox* (Von Humboldt), 1819	7	1							1	1						10
*Rh. (Run.) frontosa* (Theobald), 1903			6													6
*Rh. (Run.) reversa* Lane and Cerqueira, 1942		2	5	6		3	1		1	2	1	7	1	1		30
*Sa. (Sbn.) intermedius* Lutz, 1904			1	1			2			1	2					7
*Tr. (Tri.) digitatum* (Rondani), 1848			2			1				2						5
*Tr. (Tri.) pallidiventer* (Lutz), 1905			1													1
*Ur. (Ura.) calosomata* Dyar and Knab, 1907									35	1	5	1	3			45
*Ur. (Ura.) geometrica* Theobald, 1901									1	2		10				13
*Ur. (Ura.) lowii* Theobald, 1901									2							2
*Ur. (Ura.) nataliae* Lynch Arribalzaga, 1891									7				1			8
*Ur. (Ura.) pulcherrima* Lynch Arribalzaga, 1891					1		15				4	21	4	7		52
*Wy. (Den.) luteoventralis* Theobald, 1901			1													1
*Wy. (Pho.) edwardsi* (Lane and Cerqueira), 1942				2			1		1	1		3	3			11
*Wy. (Pho.) flabellata* (Lane and Cerqueira), 1942						1									1	2
*Wy. (Pho.) muehlensi* Petrocchi, 1927			1													1
*Wy. (Tri.) aporonoma* Dyar and Knab, 1906			2	1		4			3	2						12
*Wy. (Wye.) pertinans* (Williston), 1896			2													2
Total	143	25	54	25	25	220	245	126	1395	138	122	515	254	385	101	3773

**Table 2:** Total numbers of adult mosquito specimens collected from February 2012 to January 2014, in two sites in Guapiaçu Ecological Reserve (REGUA), Rio de Janeiro using CDC and Shannon traps.

**Species** *	**Site A**		**Site B** *	**Total**
	**CDC trap**	**Shannon**	**CDC trap**	
*Ad. (Ady.) squamipennis* (Lynch Arribálzaga, 1878)	13		2	15
*Ae. (Och.) rhyacophilus* Costa Lima, 1933	17		2	19
*Ae. (Och.) scapularis* (Rondani,1948)	53		51	104
*Ae. (Och.) serratus* (Theobald, 1901)	28		24	52
*Ae. (Pro.) terrens* (Walker, 1856)	0		16	16
*An. (Nys.) albitarsis* Lynch Arribálzaga, 1878	115		8	123
*An. (Nys.) evansae* (Brethés, 1926)	45	1	2	48
*An. (Nys.) triannulatus* (Neiva and Pinto,1922)	19	4		23
*Cq. (Rhy.) albicosta* (Peryassú, 1908)	14	28		42
*Cq. (Rhy.) fasciolata* (Lynch Arribálzaga, 1891)	48	25	27	100
*Cq. (Rhy.) juxtamansonia* (Chagas, 1907)	57	15	22	94
*Cq. (Rhy.) venezuelensis* (Theobald, 1912)	139		1	140
*Cx. (Cux.) bidens* Dyar, 1922	10			10
*Cx. (Cux.) declarator* Dyar and Knab, 1906	230		281	511
*Cx. (Cux.) usquatus* Dyar, 1918	274		299	573
*Cx. (Cux.)* sp1	99		14	113
*Cx. (Cux.)* sp2	44		6	50
*Cx. (Mel.) bastagarius* Dyar and Knab, 1906	1075		28	1103
*Li. durhamii* Theobald, 1901			17	17
*Li. flavisetosus* Oliveira Castro, 1935			15	15
*Ma. (Man.) titillans* (Walker, 1848)	196		?	196
*Ma. (Man.) wilsoni* (Barreto and Coutinho, 1944)	173		6	179
*Ps. (Jan.) ferox* (Humboldt, 1819)	8		2	10
*Ru. (Run.) frontosa* (Theobald, 1903)			6	6
*Ru. (Run.) reversa* Lane and Cerqueira, 1942	1		29	30
*Sa. (Sbn.) intermedius* Lutz, 1904	1		6	7
*Ur. (Ura.) calosomata* Dyar and Knab, 1907	35	7	3	45
*Ur. (Ura.) geometrica* Theobald, 1901	13		0	13
*Ur. (Ura.) nataliae* Lynch Arribálzaga, 1891		5	3	8
*Ur. (Ura.) pulcherrima* Lynch Arribálzaga, 1891	51		1	52
*Wy. (Pho.) edwardsi* (Lane and Cerqueira, 1942)	11			11
*Wy. (Tri.) aporonoma* Dyar and Knab, 1906			12	12
**Index**	Site A		Site B	
Total individuals collected	2873		900	
Total species observed	35		37	
Coefficient of variance (CV)	2.24		2.67	

*No specimens were collected with Shannon traps at this site.

Species totaling 5 or less individuals (number of specimens in parenthesis) found at both sites were *Ae*. (*Och*.) *fluviatilis* (Lutz, 1904) (3); *Cx*. (*Mcx*.) *imitator* Theobald, 1903 (3); and *Wy*. (*Pho*.) *flabellata* (Lane and Cerqueira, 1942) (2). Species collected only on site A were: *Cx*. (*Cux*.) *quinquefasciatus* Say, 1823 (5), *Ae*. (*Stg*.) *albopictus* (Skuse, 1895) (3); *Ma*. (*Man*.) *indubitans* Dyar and Shannon, 1925 (3); *Ur*. (*Ura*.) *lowii Theobald*, 1901 (2); *Cq*. (*Rhy*.) *chrysonotum* (Peryassú, 1922) (1). Collected only at site B: *Tr. digitatum* (Rondani, 1848) (5); *An*. (*Nys*.) *minor* Costa Lima, 1929 (2); *Wy*. (*Wye*.) *pertinans* (Williston, 1896) (2); *Hg*. (*Hag*.) *capricornii* Dyar, 1921 (1); *Hg*.(*Con*.) *leucocelaenus* (Dyar and Shannon, 1924) (1); *Tr. pallidiventer* (Lutz, 1905) (1); *Wy*. (*Den*.) *luteoventralis* Theobald, 1901 (1); *Wy*. (*Pho*.) *muehlensi* Petrocchi, 1927 (1). *Cq. chrysonotum* and *Ur. natalieae* were collected only with Shannon traps; *Cx. imitator* was collected with CDC and Shannon traps. All other specimens were collected only with CDC light traps.

**Fig 2:**
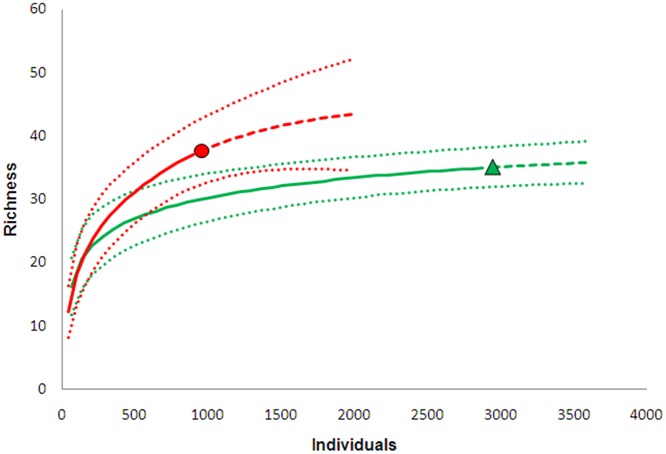
Individual-based interpolation (rarefaction; solid lines) and extrapolation (dashed lines) from reference samples from the two sampling sites (filled black circle = site A; filled gray triangle = site B) in Guapiaçu Ecological Reserve (REGUA) from a multinomial model, with 95% unconditional confidence intervals (dotted lines) (based on Colwell et al. 2012).

Species richness at each site was estimated using the total number of mosquitoes collected from each site as samples (Fig. 2 and Table 3). At site A, 35 species were observed, closely matching expected species richness based on Chao1-bc (37 species) and ACE-1 (40 species) estimates; 11.4% of the sample consisted of species for which only a single specimen was collected. At Site B, 37 species were observed, 27.0% of which were collected only once; the Chao 1-bc estimate was 43 species and that of ACE-1 51 species (Table 3). Thus, following Colwell [23] it was considered that the samples obtained provided adequate representations of species diversity at both sites. Furthermore, sampling efficiency was estimated to be 0.999 for site A and 0.989 for site B, meaning that the probability of finding additional species with further sampling was less than 1%. Colwell et al. [22] suggested that extrapolation is conservatively reliable only up to double the reference sample size. Consequently, results of rarefaction-extrapolations were compared between sites A and B at 1800 individuals, which is the double the lowest number of individuals captured at either site (site B). There was a 6.8% overlap in the 95% confidence intervals around the species accumulation curves between sites (Fig. 2). Following the conservative overlap criterion proposed by Colwell et al. [22] it was inferred that species richness did not differ significantly between sites.

**Table 3:** Mosquito diversity estimates for two sites in Guapiaçu Ecological Reserve, Cachoeiras de Macacu, Rio de Janeiro, Brazil, in the period from February 2012 to January 2014 (bootstrap mean ± s.e.; 95% confidence intervals are in parentheses).

	Site A	Site B
Diversity of order 0 (Species richness)		
ACE-1	39.8±5.2 (35.9–61.7)^a^	51.0±10.5 (40.8–88.6)^a^
Chao1-bc	36.5±2.2 (35.2–47.5)^a^	43.4±5.5 (38.5–64.3)^a^
Shannon Index (Chao & Shen 2003)	2.4±0.0 (2.4–2.5)^a^	2.2±0.1 (2.1–2.3)^b^
Shannon diversity*	11.2±0.3 (10.6–11.7)^a^	8.9±0.5 (8.0–9.8)^b^
Simpson index (MVUE)	0.17±0.0 (0.09–0.25)^a^	0.22±0.0 (0.11–0.32)^a^
Simpson diversity (MVUE)**	5.8±0.25 (5.3–6.3)^a^	4.6±0.3 (4.1–5.1)^b^

^a-b^: In each row, sites not sharing the same letter are significantly different (p<0.01).

* Diversity of order 1;

** Diversity of order 2.

**Fig 3:**
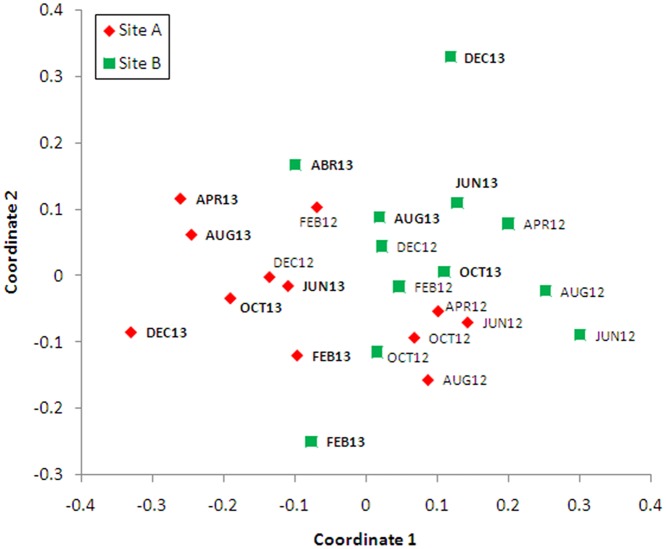
Non-parametric multidimensional scaling plot of 48 mosquito species collected bimonthly at two sites (site A represented with crosses, site B with filled circles) during 2012 and 2013. Stress is 0.22, indicating a moderately good representation of the data in the two-dimensional ordination plot. Labels indicate sample month and year.

In total, 24 species were common to both sites, 6 of which were rare (each totaling 10 or fewer individuals). CV values for each site were relatively high (Table 2), reflecting high heterogeneity in species abundances in the communities of both sites. Based on the ACE-shared model, which allows for heterogeneous discovery probabilities of species occurring at multiple sites [29–33], there are likely to be at least 4 such shared species not discovered in the survey (shared species = 27.52±11.2; 95% CI: 24.0–62.7). PERMANOVA analysis detected significant differences in species composition between sites (F = 1.91; p<0.05) but not months (F = 0.94; p = 0.57). Sites A and B were clustered separately in two dimensional ordination space when species abundances from different months and sites were analyzed using NMDS; Site A mostly grouped to the left side of the graph (Fig. 3).

GLMs assessing differences in abundance and diversity between sites showed significant interactive effects between year and site on mosquito abundance (p<0.001), species richness (as estimated with ACE-1) (p<0.004), and diversity of order 1 (p<0.001), but no significant effects of site (p = 0.37) or year (p = 0.16) on diversity of order 2. Mosquitoes were more abundant at site A than site B, and in 2013 compared to 2012 (Table 4). More species were collected at site A in 2013 and at site B in 2012. The lowest richness recorded was at site B in 2013. The effective numbers of species detected both at site B in 2012 and at site A in 2013 were approximately twice that at site B in 2013.

**Table 4:** Mosquito abundance and diversity estimates (effective number of species) for two sites in Guapiaçu Ecological Reserve, Cachoeiras de Macacu, Rio de Janeiro, Brazil, in 2012 and 2013 (adjusted ± s.e.). Diversity of Order 0 is ACE-1; Diversity of order 1 is Shannon diversity; diversity order 2 is Simpson diversity.

Site	Year	Abundance	Div. Ord. 0	Div. Ord. 1	Div. Ord. 2
Site A	2012	57.3±3.1^a^	9.2±2.5^bc^	5.4±0.9^ab^	4.9±0.9^a^
	2013	375±7.9^b^	17.5±2.5^a^	6.5±1.0^a^	4.3±0.8^a^
Site B	2012	28.8±2.2^c^	14.4±2.5^ab^	7.5±1.1^a^	4.8±0.9^a^
	2013	91.2±3.9^d^	6.5±2.5^c^	3.6±0.8^b^	2.9±0.7^a^

^a-d^: In each column, sites not sharing the same letter are significantly different (p<0.01).

**Fig 4:**
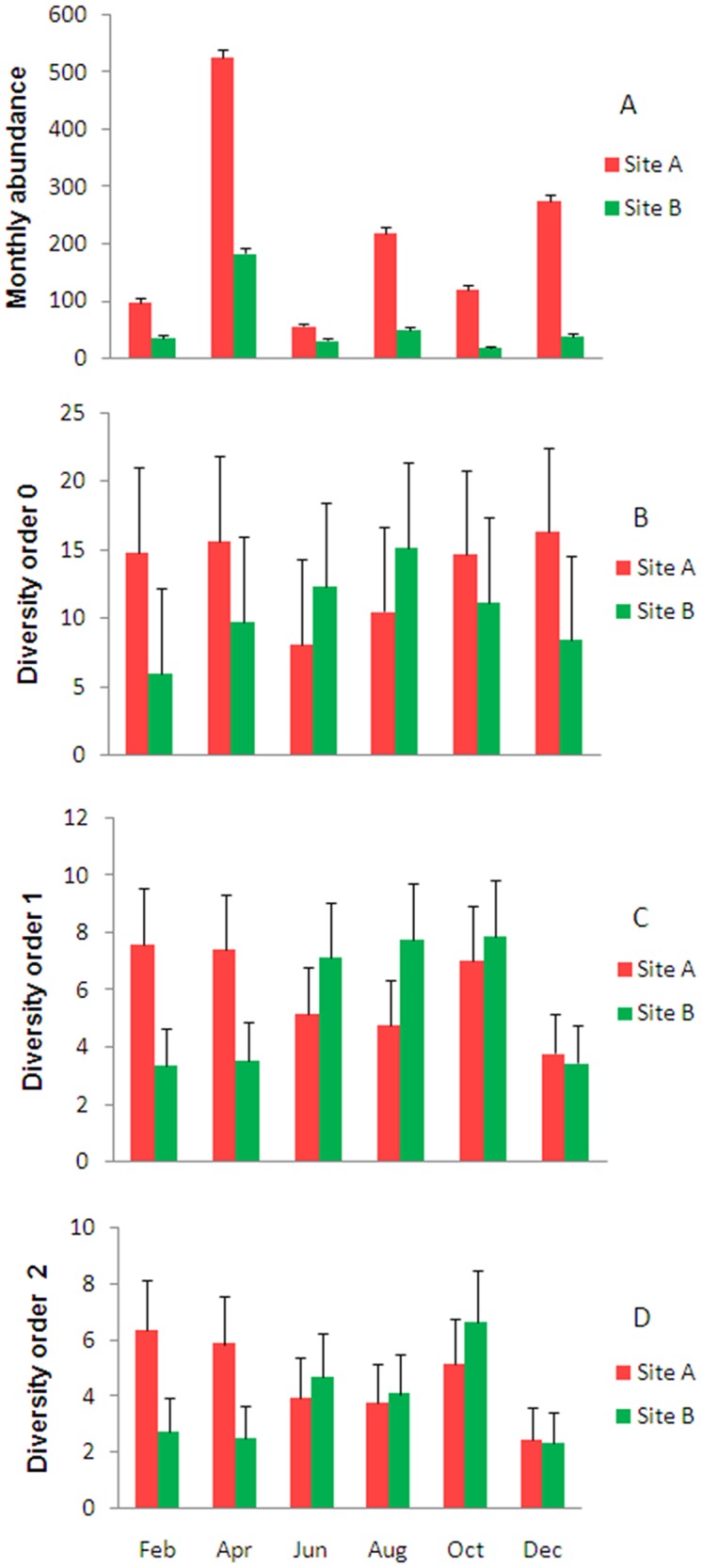
Monthly estimates of: A. species abundance and measures of diversity B. order 0 (ACE-1), C. order 1 (Shannon) and D. order 2 (MVUE) in Guapiaçu Ecological Reserve (REGUA) (adjusted means + s.e.). Different small letters indicate significant differences (p<0.01) between sites and months in mosquito abundance. No significant differences were found in measures of diversity.

There was also a significant interactive effect between month and site on mosquito abundance (p<0.001). The highest mosquito numbers were recorded in April and December, in which there were significantly more mosquitoes at site A than B. The lowest mosquito numbers were in October (site B) and June (site A). No significant effects of month or site were detected on measures of diversity (Fig. 4). Correlations between measures of diversity and climatic variables were mostly weak and non-significant: both Shannon and Simpson indices of diversity were significantly related to monthly rainfall (p<0.01 each), which was the only variable retained in these models; the models explained only 23% and 25% of the variation in the data, respectively. Mosquito abundance, on the other hand, was significantly related to monthly maximum temperature in the month prior to sampling, explaining 22% of the variation in the data (Table 5).

**Table 5:** Results of stepwise multiple linear regression analyses of mosquito abundance and measures of diversity as functions of temperature (monthly maximum and minimum), rainfall and relative humidity in the same or previous month.

Variable	Parameter	T	P
Abundance			
Tmax (prev)	0.23+0.08	2.77	0.009
Shannon diversity			
Intercept	2.15±0.13	16.07	<0.0001
Rainfall	-3.80E-03±1.30E-03	-2.81	0.009
Simpson diversity			
Intercept	1.87±0.12	15.86	<0.0001
Rainfall	-3.50E-03±1.20E-03	-2.97	0.006

Only variables with p< 0.05 were retained.

Tmax (prev) = Monthly maximum temperature of the previous month.

Stepwise linear regression analysis of variables influencing abundances of the eight most frequent species resulted in bivariate models in which minimum temperature and maximum temperature in the previous month explained 44% of the variation in *An. albitarsis* abundance, and minimum temperature in the same and previous month explained 53% of the variation in *Ma. Titillans* abundance. Forty-seven percent of the variation in *Ae. scapularis* abundance was explained by minimum temperature in the previous month, and 33% of that of *Cx. usquatus* by maximum temperature. *Cx. bastagarius* and *Ma. wilsoni* abundances were related to relative humidity in the previous month, resulting in univariate models that explained 29% and 31% of the variation, respectively. The abundances of *Cx. declarator* and *Cq. venezuelensis* were not significantly associated with any of the climatic variables measured (Table 6).

**Table 6:** Results of stepwise multiple linear regression analyses of abundances of the eight most frequent mosquito species as functions of temperature (monthly maximum and minimum), rainfall and relative humidity in the same or previous month.

Variable	Parameter	T	p
*Ae. scapularis*			
Tmin (prev)	0.54±0.17	3.24	0.007
*An. albitarsis*			
Tmax (prev)	0.33±0.12	2.78	0.02
Tmin	-0.28±0.14	-2.08	0.05
*Cx. bastagarius*			
RH (prev)	0.17±0.07	2.33	0.04
*Cx. usquatus*			
Intercept	12.43±4.03	3.09	0.009
Tmax	-0.39±0.16	-2.43	0.03
*Ma. titillans*			
Tmin (prev)	0.69±0.19	3.7	0.003
Tmin	-0.45±0.17	-2.6	0.02
*Ma. wilsoni*			
RH (prev)	0.11±0.04	2.41	0.03

Only variables with p < 0.05 were included.

Tmax = Monthly maximum temperature; Tmin = Monthly minimum temperature; RH = Relative humidity; (prev) = in the previous month.

## Discussion

The sampling procedure used provided an adequate representation of the composition of mosquito communities in REGUA, since 35 of 40 (87%) and 37 of 51 (73%) species estimated to occur at sites A and B, respectively, were detected. Overall, the number of species detected in REGUA (48 species from 14 genera) was comparable to numbers reported for other patches of Atlantic Forest, although species composition seems to vary somewhat among sites. For example, Guimarães et al. [34] collected 45 mosquito species from 13 genera in forest environments in Itaguaí, Rio de Janeiro, although only 50% of the species were the same as those collected in REGUA. In Nova Iguaçu Municipal Park, within the Gericinó-Mendanha natural protection area, also in Rio de Janeiro State, 31 species from 12 genera were reported [5], 45% of which were the same as those found in REGUA. This is consistent with the highly heterogeneous environment of the Brazilian Atlantic Forest and the associated high occurrence of endemism [35].

Even though the sampling sites were located within the same fragment of Atlantic Forest, differences in the available oviposition sites may explain differences in the mosquito fauna. Although species richness was similar, total diversity was higher at site A than site B. Species composition and abundance differed, with approximately 25% of species common to both. Ground water mosquito species (such as *Cx. bastagarius*) and species of the Mansoninii tribe were found almost exclusively at sampling site A. Site A was near a lake with calm, clear, cold water, a low light environment, and plenty floating and emergent vegetation, such as *Eichhornia* spp. It thus offered ample larval habitat for these species throughout the study period. On the other hand, species of the Sabethini tribe, mosquitoes that are typically sylvatic, were more frequently found at site B, characterized by more extensive plant cover and mature forest.

Several hypotheses have been proposed to explain differences in species richness and diversity. One such is the niche diversification hypothesis, in which diversity is a function of the range of habitats and of the degree of specialization of resident species; in this hypothesis more stable ecosystems such as forests are predicted to have higher species diversities. The intermediate disturbance hypothesis (IDH) states that local species diversity is maximized when ecological disturbance is neither too rare nor too frequent, allowing both competitive and opportunistic species to coexist [36]. This study did not find significant differences in species richness between the two Atlantic Forest sites analyzed. However, Site B, the more intact environment, had lower diversity (relating richness with relative abundance) and greater dominance of Culicidae, which could be explained in part by the IDH. Differences in larval habitat availability and/or preference may also explain the patterns observed. Those species of Culicidae that develop in water-filled tree holes and other phytothelmata such as the Sabethini tribe (with the exception of *Limatus durhamii*, which has been collected from habitats such as artificial containers [37] and percolation tanks [38]) were more frequently collected in Site B (43% compared with 8% in site A). Such species are considered to indicate environments that have not been subjected to pronounced anthropic disturbance [39]. In contrast, 91% of mosquitoes collected at Site A develop in larval habitats such as ground pools, ponds, and artificial containers; the presence of specimens of the Mansoniini tribe (*Mansonia* spp., *Coquillettidia* spp.) and the Aedini tribe (e.g., *Ae. scapularis*) tends to be linked to environments with higher degrees of anthropic disturbance [39], [19].

Measures of diversity showed little variation between years and no significant variation among months, and were weakly correlated with climatic variables. Although relationships with rainfall were significant, these explained less than 30% of the variability of the data. However, it is interesting that the relationship was negative, which could indicate that a few species peak in numbers as rainfall increases (however, no correlation was found between rainfall and the abundances of the most common species, see below). Alternatively high rainfall may be “flushing” larvae from their habitats. Dorvillé [39] pointed out that in some regions of Brazil there are alternations between tropical and temperate climatic states [40]. In our study sites average temperatures during the sampling period fluctuated between 20 and 26°C and mode monthly rainfall was 50 mm, indicative of a tropical climate suitable for mosquitoes year round, rather than a temperate one. Thus factors other than climate may have more influence on temporal variations in the mosquito community.

*Aedes scapularis* is a flood water mosquito; its eggs hatch in installment in response to floods. Rainfall regime directly influences the development of immature *Ae. scapularis* [41], [19], which has been suggested as an explanation for higher occurrences in warm, humid periods with heavy rainfall [42] for this species and *Ae. albifasciatus* [43], [44]. In this study no significant relationships were found between *Ae. scapularis* abundance and rainfall, but there was a positive relationship with average minimum temperature in the previous month. This species was collected in similar numbers at both sites, reflecting the generalist nature of *Ae. scapularis* reported by Forattini et al. [45] in São Paulo State, as well as Lourenço-de-Oliveira and Silva [46] and Guimarães et al. [34] in Itaguaí-Rio de Janeiro. *Aedes scapularis* is a vector of *Dirofilaria immitis* Leidy in Rio de Janeiro State [47], and there is field and laboratory evidence indicating it may be a vector of several arboviruses [19].

Overall, *Culex bastagarius* was the most abundant species collected, although it was comparatively rare in 2012. The species is a suspected vector of *Hepatozoon caimani* (Apicomplexa: Hepatozoidae) in some regions of South America [48]. Luz & Lourenço-de-Oliveira [49] reported that *Culex bastagarius* abundances are lower during the rainy season. However, in this study no significant relationship was found between *C. bastagarius* abundance and rainfall; however a positive (but weak) relationship was detected with relative humidity in the previous month, and the species was very abundant during the wetter months. Regression models seeking to explain abundances of six of the eight most frequent species in terms of climatic variables explained from 29 to 53% of the variation in the data, suggesting that local conditions other than climate, such as interspecific interactions, may have a substantial influence on seasonal fluctuations in abundance.

Culicid species composition differed between the environments studied, probably influenced by the intactness of the forest and other local characteristics such as larval habitat availability. Among the species considered of epidemiological importance, *Mansonia* and *Coquillettidia* species, prevalent at Site A, are known vectors of equine encephalitis [19]. *Hg. leucocelaenus*, collected only at Site B (though in small numbers), is involved in the transmission of yellow fever [50], and potentially of Ilhéus, Maguari, Tucunduba, and Una viruses [51]. Other species of medical interest such as *Ae. scapularis* and *Cx. declarator* (a vector of Saint Louis Encephalitis in the Amazonian region), [52] were detected in similar numbers at both sites.

Due to the diversity of mosquito species detected, their potential roles in pathogen transmission, and the intermittent flow of national and international visitors to REGUA, it is recommended that a program of Culicidae surveillance be maintained.
